# Books Reviewed

**Published:** 1865-01

**Authors:** 


### BOOKS REVIEWED.

*Diphtheria; its Nature and Treatment, with an account of the History of its Prevalence in various countries. By* DANIEL D. SLADE, M. D., *being a second and revised edition of an Essay to which was awarded the Fiske Fund Prize* 1860. Philadelphia: BLANCHARD & LEA, 1864.

To show the character of this essay, we quote entire the author's *resume:* "In the preceding pages, we first gave Bretonneau's description of diphtheria. We then remarked that it was only by a comparison of the various epidemics of 'sore throat' which had prevailed at intervals in various parts of the world, that we could ascertain how far his description was to be taken as a model of the disease. Accordingly we took up the history from remote ages to the present day.

"Having given an account of those which had prevailed in various parts of Europe, and having compared the descriptions of various writers upon these epidemics, with that of Bretonneau, we showed that he was incorrect in denying the presence of all constitutional disturbance, as also in insisting upon the absence of all relation between diphtheria and gangrene of the fauces—both of these conditions having been frequently observed, particularly during the epidemics of late years.

"We next observed that Bretonneau's idea of croup, which he associates with diphtheria, does not conform to our ideas of that disease. founded, as they are, upon the description given by Dr. Home. The distinctions between diphtheria and croup were dwelt upon, as also the non-identity of diphtheria and scarlatina.

"In order, we took up the history of the disease in England. A comparison of the descriptions of the disease by various writers, as it appeared in the several counties, gave no marked uniformity, and but little correspondence with Bretonneau's model.

"Diphtheria in America was then considered, and we gave at some length a description of an epidemic of 'sore throat,' by Dr. Bard, also an account of the epidemics in California and other parts of the Union.

"We next remarked that all these epidemics of 'sore throat' were connected by a bond of union, to be found in the pathological anatomy of the disease, which consists in the peculiar exudation. That although Bretonneau fully recognized this fact, his description was deficient, hence we subjoined that of MM. Barthez and Rilliet, as being more comprehensive. We also considered the disease as existing under two forms, the mild and severe.

"Certain points as respects the nature of the disease were taken up in order. First, the characteristics of the false membrane, its physical appearances, its seat, the experiments of Bretonneau in order to ascertain the specific nature of the diphtheritic membrane, its microscopic appearances, and its dependence upon certain parasites were discussed.

"Next, in answer to the question, Is dipththeria infectious? Having given the arguments of various authors, we replied, that although we were ignorant of the exact laws which governed these epidemics, we are able to detect certain hygienic or individual circumstances which undoubtedly had their effect, either as direct or as pre-disposing causes.

"The presence of albumen in the urine and its signification were commented upon. We remarked that further observation was necessary before we could ascribe to it any settled prognostic value. We spoke of the singular after-effects of the disease, as shown especially upon the nervous system. We gave the observations of MM. Trousseau, Faure, and others upon this point.

"In our account of the *treatment* of diphtheria, we said that it was only within the last few years that anything like unanimity had prevailed. That it was now universally regarded as an asthenic disease, and consequently would bear no depletory measures, but, on the contrary, required tonics, stimulants, and a nourishing diet, even in the early stages. Blisters, leeches, and local bleeding of any sort should be prohibited.

"The tonics best suited we enumerated. Of the auxiliary measures, we first spoke of the local applications to the fauces, their utility and propriety, the various agents which had been employed and the mode of use. The ablation of the tonsils recommended by Bouchut, we conceived to be inadmissible, excepting under rare circumstances.

"Tracheotomy we discussed at considerable length. Remarking that the two diseases, inflammatory croup and diphtheria, were on an equal footing as regards the applicability of the operation, we answered the various objections which had been brought against it; the small amount of success; the difficulties of performing it; the tendency to the production of bronchitis, &c. * * *

"We considered that the proper time for performing the operation was an intermediate period. We gave some necessary rules as to the size of the canula, the state of the surrounding atmosphere, the importance of having some competent person at hand in the case of emergency, the propriety of keeping up the medical treatment, and the time for removing the canula.

"Having given a summary of the treatment recommended by some of the leading men in Europe, we concluded by a brief consideration of the operation for 'tubing the larynx.'"

---

*Laryngoscopal Medication; or, the Local Treatment of the Diseases of the Throat, Larynx and neighboring organs, under sight. By* Louis Elsberg, A. M., M. D., *Lecturer on the Diseases of the Larynx and Throat in the University of New York. New York:* Wm. Wood & Co., 61 *Walker street,* 1864.

We make the following quotations from the book:

"How exceedingly simple, therefore, is the principle on which laryngoscopy is founded! In the words of Czermak, the eminent professor of physiology in the University of Prague:

'A small, flat mirror, with a long stem, previously warmed, to prevent its being tarnished by the breath, is introduced into the mouth, widely open, as far as its back part. It is then held up in such a manner as to permit the rays to penetrate it, on the one hand, and consequently to illuminate those parts which it is desirable to examine; and on the other hand, the image of those parts is reflected into the eye of the observer.'

"The individual parts revealed by the laryngoscope, which are otherwise completely invisible or rarely or never seen without difficulty, are: the postero-inferior portion of the base of the tongue; the glossal insertion and posterior surface of the glosso palatine arches; the glosso-hyoid folds, or lateral glosso-hyoid ligaments; the glosso-epiglottic ligament; the valleculæ; the pharyngo-palatine arches with the vestibulum pharyngis medium; the pharyngo-epiglottic ligaments; the lateral hyo epiglottic ligaments; the epiglottis, *i. e.* its free anterior surface, lateral and upper borders and crest, and whole posterior surface; the capitulum of the hyoid bone; the aryteno-epiglottic, or as they should be named more briefly 'ary'-epiglottic, folds; the cuneiform cartilages; the supra-arytenoid cartilages; the arytenoid cartilages; the inter-arytenoid fold; the pyriform sinuses; the posterior wall of the pharynx down to its attachment to the cricoid and arytenoid cartilages; the upper cavity of the larynx with all its anatomical relations and contents; a portion of the lower cavity of the larynx, *i. e.* particularly its anterior wall; aud the anterior wall, and sometimes lateral walls of the trachea for a more or less considerable distance down—under favorable circumstances down to the bifurcation, and in a few instances, placed on record by able observers, even throughout the whole length of the right bronchus.

"The posterior part of the mucous membrane of the intercartilaginous rima glottidis, and the posterior and lateral portions of the lower laryngeal cavity, as well as the lower portions of the trachea, aad further down, can usually not at all, or but imperfectly be seen.

"With the further addition of a little hook, to hold, as occasion requires, the uvula and soft palate out of the way, the apparatus described for laryngoscopy reveals, on mediate inspection in the same little mirror—previously warmed as before, and now turned upwards—the posterior surface of the velum and upper posterior wall of the pharynx, the orifices of the Eustachian tubes, and Rosenmüller's fossa, the posterior nasal septum and nares, the turbinated bone and turbinated processes of the ethmoid bone, in short, the whole pharyngo-nasal space (bounded on the sides by the pterygoid processes of the sphenoid bone; in front by the nasal fossæ, above by the body of the sphenoid bone, and behind by the basilar portion of the occipital bone and upper cervical vertebræ,) under favorable circumstances, to the covering mucous membrane of the nasal bones, and of the plate of the ethmoid.

"If, instead of the palate-hook, we add to our little mirror and other apparatus a proper forceps, we have the outfit for œsophagoscopy. This renders the dilatation of the œsophagus necessary, and although I have not yet succeeded in obtaining a good view into the œsophagus, except at its commencement, yet that œsophagoscopy can be accomplished, even if but to a limited extent, has been proved by the practice of Lewin in Berlin, and Semeleder in Vienna."

We hope that no one of our readers will for a moment believe that the laryngoscope can be made to show what this paragraph would seem to intimate. It is to be regretted that a really valuable instrument should be misrepresented in this way; it is wrong to attempt any such imposition upon the profession. The laryngoscope will show surfaces beyond inspection without it; is a valuable instrument and should be highly estimated; but its value should not be so over-stated as to disgust all honest seekers after the truth. This representation of its scope is made by unnecessary and pedantic division of surfaces much greater than it really is, but it is still greatly over-stated. The book should be carefully perused; it contains many vauluable suggestions and is in the main exceedingly instructive.

---

*Man and his Relations: Illustrating the influence of the mind on the body; the relations of the faculties to the organs and to the elements, objects and phenomena of the external world.* By S. B. Brittan, M. D., *New York:* W. A. Townsend, 1864.

We have copied the title page of this volume entire in order to give the author's own statement of what he would be at. We have given the work a somewhat careful examination, but must confess that the hopes we had formed from its title were not borne out by the contents. The writer is a believer in Animal Magnetism, Spiritualism, *et id genus omne* and has written a work in their behalf. Now be it understood, we do not in the least object to any man holding these views, if he choose, but if he put them forth as scientific facts and attempt to prove them by reference to scientific reasoning, he must consent to be judged by the same rules which govern us in our judgment of other scientific works, and judged in this way we are constrained to call the work before us a failure; not however because it advocates doctrines which we do not believe, for we are perfectly ready to be convinced of the truth of any or all the author's tenets, provided it be done through the medium of our reasoning faculties, and not through our imagination; but because he does not give us logical deductions from ascertained facts.

To do our author justice we must say that we do not know that he intended to convince any unbelievers of the truth of his peculiar doctrines; he seems rather to assume their truth as already proven, and to have intended to present, from that stand-point, Man's relations to this world and the regions beyond ; and we presume to fully appreciate the work one should first become a believer in the views of its author. In its style the book is somewhat "sophomorical" for a purely scientific and philosophical treatise, and contains rather more raphsody than reasoning; but we have neither space nor inclination to enter into any discussion upon its truth or falsehood.

The typhographical execution of the work is excellent, the type clear, and the portrait of the author prefixed to the volume is a really fine specimen of steel engraving. P.

*The Army Surgeon's Manual, for the use of Medical Officers, Cadets, Chaplains, and Hospital Stewards, containing the Regulations of the Medical Department, all General Orders from the War Department, and Circulars from the Surgeon-General's Office, from January* 1*st*, 1861 *to July* 1*st*, 1864. *By* WILLIAM GRACE, *of Washington, D. C. Published by permission of the Surgeon-General. New York:* BAILLIERE BROTHERS, 520 *Broadway*, 1864.

*Part* 1*st*—Contains list of the staff U. S. Army, July 1st, 1864.

*Part* 2*d*—Regulations of the Medical Department, from the Revised Regulations for the Army.

*Part* 3*d*—General Orders relative to the Medical Department.

*Part* 4*th*—Circulars, etc., etc.

There is a very full index, which adds very much to the value of this book. Surgeons and assistant surgeons however well qualified for their professional duties, will find many things required of them in entering the army with which they are quite ignorant. This manual is designed as a guide, and we think that every surgeon would act wisely in taking it as a "pocket companion." He will find his duties very fully defined, and will be instructed in what he most wants to know.

*Report of a Successful Operation in a case of Subclavian Aneurism, by* A. W. SMITH, M. D., *House Surgeon, Charity Hospital, New Orleans, Louisiana.*

This report is inscribed by the author to Valentine Mott, M. D., who first performed the operation of ligaturing the innominate artery. It is a report of the first successful operation. This success he attributes to the ligation of the vertebral artery, thus preventing retrograde circulation. This operation was made fifty-four days after the ligation of the innominate artery, and on account of hæmorrhage, which could not otherwise be controlled. The report and the results of the operation reflect great credit upon the surgical skill of the author.

---

*Transactions of the Medical Society of the State of Pennsylvania, at its Fifteenth Annual Session, held in Philadelphia, June,* 1864. *Third Series—Part* 3. *Published by the Society.*

The fifteenth annual session of the Medical Society of the State of Pennsylvania was opened by an address from the president, Dr. Wilson Jewell upon the early history of the principal Medical Institutions of Philadelphia.

Reports were received from a few of the County Societies, furnishing a partial history of the sanitary condition of the state. From this it appears that *typhoid fever* has been quite prevalent throughout the state. In some counties *bilious fever* was reported prevalent with tendency to congestion of the brain and lungs, in other counties there was what is called *mixed fevers*, by which is meant a remittent typhoid fever. All such cases recovered. Scarlet fever is also reported as prevalent. Notices of the occurrence of *spotted fever* were received from Chester, Montgomery, Northampton, Philadelphia, Susquehanna and Westmoreland counties. It is represented as exceedingly fatal; its close resemblance to cerebro-spinal meningitis is remarked in all reports of the disease. In the treatment almost everything has been favorably mentioned; "heroic" treatment had generally been adopted. Diphtheria, Erysipelas, Small Pox and Measles were also represented as having prevailed extensively in some localities. The volume of Transactions is made up mostly of the reports of the county societies. The Constitution and By-Laws of the society is also published, and a list of the presidents, permanent members &c. &c.

---

